# Selenoprotein P as Biomarker of Selenium Status in Clinical Trials with Therapeutic Dosages of Selenite

**DOI:** 10.3390/nu12041067

**Published:** 2020-04-12

**Authors:** Ola Brodin, Julian Hackler, Sougat Misra, Sebastian Wendt, Qian Sun, Elena Laaf, Christian Stoppe, Mikael Björnstedt, Lutz Schomburg

**Affiliations:** 1 Department of Laboratory Medicine, Division of Pathology F46, Karolinska Institutet, Karolinska University Hospital Huddinge, SE-141 86 Stockholm, Sweden; ola.brodin@sll.se (O.B.); som058@mail.usask.ca (S.M.); 2 Department of Head and Neck, Lung and Skin Cancer, Karolinska University Hospital, SE-17176 Stockholm, Sweden; 3 Institute for Experimental Endocrinology, Charité-Universitätsmedizin Berlin, Corporate Member of Freie Universität Berlin, Humboldt-Universität zu Berlin, and Berlin Institute of Health, Augustenburger Platz 1, CVK, D-13353 Berlin, Germany; julian.hackler@charite.de (J.H.); qian.sun@charite.de (Q.S.); 4 Department of Intensive Care Medicine, RWTH Aachen University, D-52074 Aachen, Germany; swendt@ukaachen.de (S.W.); elaaf@ukaachen.de (E.L.); cstoppe@ukaachen.de (C.S.)

**Keywords:** supplementation, trace element, monitoring, chemotherapy, adjuvant treatment

## Abstract

Selenoprotein P (SELENOP) is an established biomarker of selenium (Se) status. Serum SELENOP becomes saturated with increasing Se intake, reaching maximal concentrations of 5–7 mg SELENOP/L at intakes of ca. 100–150 µg Se/d. A biomarker for higher Se intake is missing. We hypothesized that SELENOP may also reflect Se status in clinical applications of therapeutic dosages of selenite. To this end, blood samples from two supplementation studies employing intravenous application of selenite at dosages >1 mg/d were analyzed. Total Se was quantified by spectroscopy, and SELENOP by a validated ELISA. The high dosage selenite infusions increased SELENOP in parallel to elevated Se concentrations relatively fast to final values partly exceeding 10 mg SELENOP/L. Age or sex were not related to the SELENOP increase. Western blot analyses of SELENOP verified the results obtained by ELISA, and indicated an unchanged pattern of immunoreactive protein isoforms. We conclude that the saturation of SELENOP concentrations observed in prior studies with moderate Se dosages (<400 µg/d) may reflect an intermediate plateau of expression, rather than an absolute upper limit. Circulating SELENOP seems to be a suitable biomarker for therapeutic applications of selenite exceeding the recommended upper intake levels. Whether SELENOP is also capable of reflecting other supplemental selenocompounds in high dosage therapeutic applications remains to be investigated.

## 1. Introduction

The essential trace element selenium (Se) is needed for the biosynthesis of selenoproteins and, therefore, is of fundamental importance for human health [1]. An insufficient daily intake causes a low Se status, characterized by a marginal expression of selenoproteins [2]. Certain tissue- and gene-specific mechanisms ensure that the most essential organs like the brain and endocrine tissues remain preferentially supplied in times of low intake, and that the most essential selenoproteins are preferentially biosynthesized [3]. Population-wide Se deficiencies are observed in certain areas of Africa and Asia and have been associated with three endemic and Se-responsive diseases, that is, myxedematous cretinism [4], Kashin–Beck disease [5], and Keshan disease [6]. European populations are considered as only moderately supplied, and low Se status has been described as a risk factor for certain diseases, for example, cancer at several sites, autoimmune thyroid, or cardiovascular disease [7,8,9]. More than one biomarker has been determined in a subset of these studies, where low Se status was related to disease risk, disease severity, or odds of convalescence, for example, in studies of hepatocellular [10], colorectal [11], or prostate cancer [12]; traumatic brain injury [13]; neonatal infection [14]; or genetically impaired selenoprotein expression [15]. In most of these analyses, the three Se status biomarkers—total serum Se concentration, plasma glutathione peroxidase (GPX3) activity, and level of the transport protein selenoprotein P (SELENOP)—accorded well and displayed a high correlation, particularly in studies with marginally supplied subjects. For reasons of stability, response range, and standardization, total serum or plasma Se and SELENOP concentrations, respectively, appear as the most reliable and meaningful Se status parameters [2,16,17].

In Se supplementation studies, SELENOP constitutes a particular suitable biomarker of Se status, and its concentration increases over a wider range of Se intakes than GPX3 [16,18,19]. However, similar to GPX3, circulating SELENOP shows saturable kinetics in time-resolved analyses or dosage escalation trials, reaching maximal expression levels of 5–7 mg SELENOP/L [2,16,20]. The Se dosages applied in these kinetic or dose escalation studies were typically below the tolerable upper intake level (UL), that is, below 400 µg Se/day [21]. Yet, some populations are at a constant supply exceeding this recommended threshold [22], and clinical trials using Se for therapeutic purposes sometimes apply higher dosages, especially in the intensive care unit (ICU), when trying to support patients with systemic inflammatory response syndrome or sepsis [23,24,25,26]. A reliable biomarker of Se status for such therapeutic intakes exceeding the UL is missing, and no suitable candidate has been suggested [2]. We speculated that therapeutic selenite administrations exceeding the UL may nonetheless be mirrored in increasing circulating SELENOP concentrations, and that 5–7 mg SELENOP/L do not yet constitute the absolute maximum. In order to test this hypothesis, samples from two separate high dosage trials with i.v. applications of selenite were analyzed. Our data support the notion of circulating SELENOP as the long sought biomarker for therapeutic Se applications in a clinical setting.

## 2. Materials and Methods 

### 2.1. Clinical Samples

Samples from two clinical trials employing high dosage selenite treatment were analyzed in this study, that is, from the *Selenite in the Treatment of Patients with Carcinoma* (SECAR) phase I trial in cancer patients [27], and from the *High-dose Sodium Selenium Supplementation in Patients With Left Ventricular Assist Device* (SOS-LVAD) study of cardiac patients with end-stage heart failure undergoing surgery for implementation of a ventricular assist device (ClinicalTrials.gov, Identifier: NCT02530788). The trials obtained ethical approval by the respective Ethical Committees of Stockholm (ethic vote 2006/429-31/3) and RWTH Aachen University (ethic vote EK 249/13), respectively. All patients provided informed written consent prior to enrollment, and the studies were conducted in accordance with the principles of Helsinki.

### 2.2. SECAR Study Design

**Figure 1:**
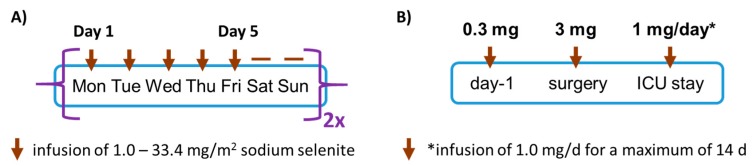
Clinical studies involving intravenous selenite treatment. (**A**) In the *Selenite in the Treatment of Patients with Carcinoma* (SECAR) phase 1 clinical study, cancer patients received an infusion of different dosages of sodium selenite once per weekday, with a break at the weekend, for two or four cycles. Plasma samples were taken before infusion and at day 5. (**B**) In the SOS-LVAD study, patients received placebo or 300 µg of selenite the day before surgery, placebo or 3 mg of selenite after induction of anesthesia before surgery, and placebo or 1 mg of selenite (*) directly after completion of surgery and daily during the stay on the intensive care unit (ICU) for a maximum of 14 days. Plasma samples were collected at each time point prior to infusion with placebo or selenite.

The SECAR phase I trial is an open-label dose-escalation study with sodium selenite (Intro-Selen i.v., Pharma Nord ApS, Vojens, Denmark) as single agent [27]. In total, 34 patients with different malignancies were enrolled [27]. Plasma samples of a subset (*n* = 9 males and *n* = 12 females, age range (median (interquartile range, IQR); 62 (59.0, 65.5) y) were analyzed in this study. Each treatment group consisted of three to six patients receiving the same daily dosage starting with 0.5 mg Se/m^2^ (i.e., 1.1 mg of sodium selenite per square meter per day). If intolerable toxicity was not observed, the next patient received a higher dosage according to a prefixed dose escalation schedule with the following amounts: 1, 1.5, 2, 3, 4.5, 6.8, 10.2, 12.8, and 15.3 mg Se/m^2^. If 1/3 of the patients had intolerable toxicity, three more patients were included, as described [27]. If 2/3 or 2/6 of the patients had intolerable toxicity, this dosage level was considered too high and the former dose level was considered the maximal tolerated dose (MTD). Unfortunately, not all blood drawings were conducted as scheduled for different reasons, for example, treatment break for the occurrence of intolerable toxicity, on behalf of the patient’s own will, because of HIV infection, or because of unsuccessful attempts to get a blood sample. The treatment groups received 10 treatments during two weeks (no treatment during weekends) (Figure 1A). Blood was collected 5 min prior and post infusion, and plasma was isolated and stored at −80 °C until analyses.

### 2.3. SOS-LVAD Study Design

The SOS-LVAD study enrolled cardiac surgery patients scheduled to undergo implantation of a ventricular assist device for the hemodynamic support of a failing heart. In total, 21 patients were assessed for eligibility and randomly assigned to one of the treatment groups. One patient was lost for follow-up, so that 10 versus 10 patients were treated with Se and placebo, respectively. A set of samples covering different time points of this intervention study from almost half of the patients (*n* = 9) was available for this analysis. Patients in the intervention group received 300 µg of sodium selenite (Selenase^TM^ i.v., Biosyn GmbH, Fellbach, Germany) the evening before surgery, followed by a high dose of intravenous selenite supplementation (3.0 mg after induction of anesthesia, 1.0 mg after surgery, and 1.0 mg daily during their ICU stay for a maximum of 14 days) or placebo (Figure 1B). Plasma samples were collected at each time point, that is, prior to the supplementation, before (baseline) and during surgery, and thereafter.

### 2.4. Selenium Measurement and SELENOP Quantification by ELISA and Western Blot Analysis

Plasma Se concentrations were quantified by total reflection X-ray fluorescence (TXRF), as described previously [11,17]. SELENOP concentrations were measured by a validated commercial SELENOP-specific ELISA (selenOtest^TM^, selenOmed GmbH, Berlin, Germany) as described [28]. For Western blot (WB) analysis, standard curves were generated using diluted NIST 1950 reference plasma (National Institute of Standards and Technology, Gaithersburg, MD 20899, USA) with known SELENOP values [28,29]. A commercial mouse monoclonal antibody (Selenoprotein P, B-9, Catalog # sc-376858, dilution; 1:500) and an in-house antibody (dilution 1:2000 [28]) were used for SELENOP detection, in combination with horseradish peroxidase-conjugated secondary antibody (Dako rabbit anti-mouse, 1:3000). Samples (0.1 µL of NIST 1950 plasma or patient sample) were separated by 12% SDS-PAGE (Bio-Rad, Stockholm, Sweden), and transferred to a 0.20 µm pore-sized PVDF membrane (Bio-Rad). Membranes were incubated with primary antibodies diluted in 5% milk overnight at 4 °C, washed three times with TBST (20 mM Tris, 150 mM NaCl, 0.1% Tween-20), incubated with secondary antibody diluted in 5% milk for 1 h at room temperature (RT), and developed with enhanced chemiluminescence (WesternBright Sirius HRP substrate, Advansta, San Jose, CA, USA). Digital images were acquired using LiCor Odyssey Fc imaging system, and quantification of immunoreactive bands was carried out by Image Studio Lite (version 5.2).

### 2.5. Statistical Analysis

Statistical analysis was performed with the Statistical Package for the Social Sciences (SPSS Statistics 21^®^, IBM, Chicago, IL, USA) and GraphPad Prism software (Version 7, GraphPad Software Inc., San Diego, CA, USA), respectively. Normal distribution of values was assessed by the Shapiro–Wilk test. Paired two-tailed t-tests were applied to compare subjects before and after treatment. The relationship between parameters was tested by Pearson’s correlation analysis. For not-normally distributed variables, the Mann–Whitney U-test, the Wilcoxon-test, and the Spearman’s correlation test were used. The quantified Western blot signals were analyzed using nonparametric tests. Linear regression analysis was conducted to specify associations of variables. The results were considered as statistically significant when the *p*-value was less than 0.05, and differences are marked as follows: *p* < 0.05 (*), *p* < 0.01 (**), and *p* < 0.001 (***). Parametric data are represented as means ± standard deviation (SD), and nonparametric data by medians and interquartile range (IQR).

## 3. Results

### 3.1. Correlation of Plasma Selenium and SELENOP Levels Before and After Supplementation

**Figure 2:**
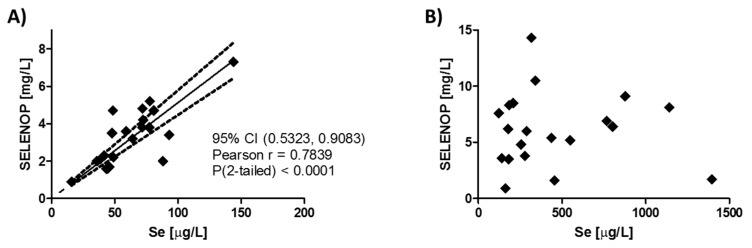
Se status at baseline and after four days of selenite infusion in the SECAR study. (**A**) Plasma Se and selenoprotein P (SELENOP) levels were linearly correlated at baseline, that is, at day 1 before supplementation. (**B**) The two biomarkers of Se status showed no correlation after four daily selenite infusions on day 5. Pearson’s correlation analysis; *n* = 21 data pairs (left) and 20 data pairs (right), respectively. CI, confidence interval.

The median Se status of the subjects enrolled into the SECAR study at baseline was 3.4 mg SELENOP/L (IQR; 2.0–4.5 mg/L), and 58.8 µg Se/L (IQR; 43.8–77.5 µg/L), respectively. These values are lower than the average Se status of healthy Europeans, who displayed concentration of 84.8 µg Se/L and 4.4 mg SELENOP/L, respectively, in a large cross-sectional analysis of almost 2000 subjects [11]. The plasma SELENOP and Se concentrations showed a linear positive association (Pearson r = 0.7839, 95% confidence interval (CI) (0.5323, 0.9083), *p* < 0.0001) at baseline (Figure 2A), but not after four days of intravenous selenite infusion, that is, after high dosage Se intake (Figure 2B).

### 3.2. SELENOP Increases in Cancer Patients in Relation to Time, Selenite Dosage, Age, and Sex

**Figure 3:**
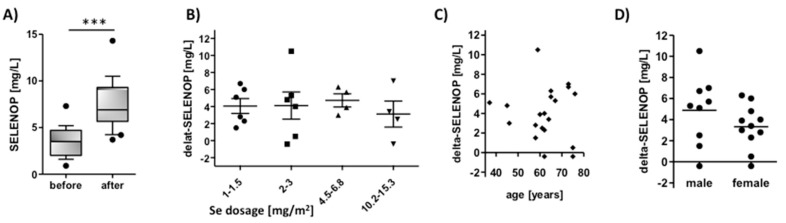
Increase of Se status biomarkers after four days of selenite infusions in the SECAR trial in relation to time, dosage, age, and sex. Cancer patients received different amounts of Se (i.v.) daily, ranging from 1 to 33.4 mg selenite/m^2^. (**A**) SELENOP concentrations increased strongly from baseline on day 1 (before) to day 5 (after) daily infusions. Incremental SELENOP increase was independent of (**B**) Se dosage infused, (**C**) age, or (**D**) sex of the patient. Analysis of SELENOP levels before and after supplementation were done by paired two-tailed *t*-test; ***, *p* < 0.0001.

SELENOP constitutes an established biomarker of Se status, and circulating SELENOP levels are elevated upon supplemental Se in clinical trials, allowing a tight monitoring of compliance [30]. Following the i.v. administration of therapeutic dosages of selenite in severely sick cancer patients of the SECAR trial, SELENOP levels increased strongly to values exceeding the expected maximal level reported in prior studies, that is, to concentrations of >7 mg SELENOP/L (Figure 3A). The incremental increase in SELENOP (difference of SELENOP before and after selenite infusion) was on average in the range of 4 mg/L, and independent of the selenite dosage infused (Figure 3B), age (Figure 3C), or sex (Figure 3D) of the patients. Individual responses are provided as supplement (Supplementary Figure S1).

### 3.3. Validation of ELISA-Based SELENOP Quantification Results by Western Blot Analysis

**Figure 4:**
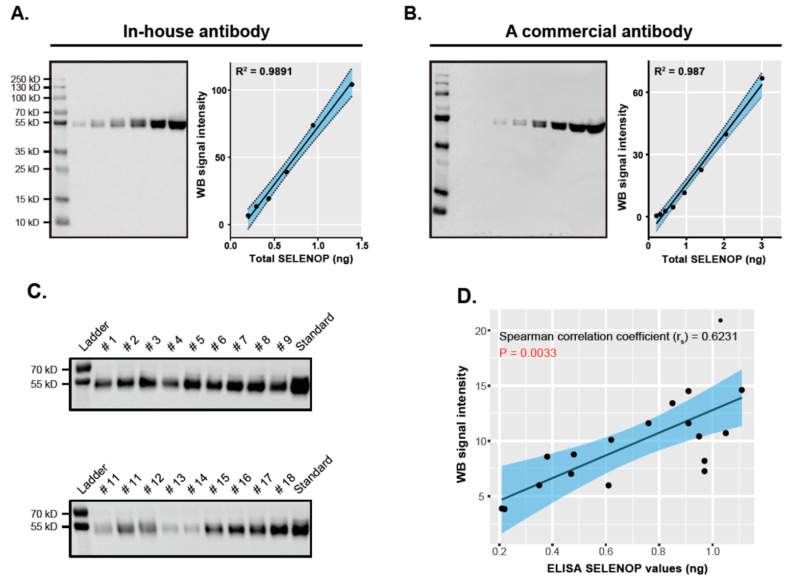
Validation of ELISA results by Western blot (WB) analyses using two independent antibodies. Plasma samples from the NIST1540 reference sample were used for optimizing and standardizing SELENOP quantification by Western blot with (**A**) an in-house and (**B**) a commercial antibody. (**C**) Western blot-based analysis detected the typical SELENOP band pattern. (**D**) Western blot results correlated over the full concentration range with the SELENOP values determined by ELISA.

The SELENOP concentrations determined by ELISA in the Se-treated cancer patients were unexpected, and some were exceptionally high. In order to validate the data and test for potential SELENOP isoforms that may have affected the quantification by ELISA, a commercially available and an in-house SELENOP-specific monoclonal antibody were used in Western blot analyses of patient samples and the NIST1540 reference plasma with defined SELENOP content. The intensities of the Western blot signals increased in proportion to the SELENOP amounts applied, irrespective of the antibody used (Figure 4A,B). The typical pattern of two adjacent SELENOP bands at around 55 kD was detected in both analyses, consistent with the predicted molecular weight of SELENOP [31]. Testing a set of representative plasma samples from the Se-treated patients, correctly sized and distinct bands of SELENOP were detected by both antibodies, and the signal strengths correlated positively and linearly to the results determined by ELISA (Figure 4C,D).

### 3.4. Response of SELENOP Levels to High Dosages of Intravenous Selenite in Cardiac Surgery Patients

**Figure 5:**
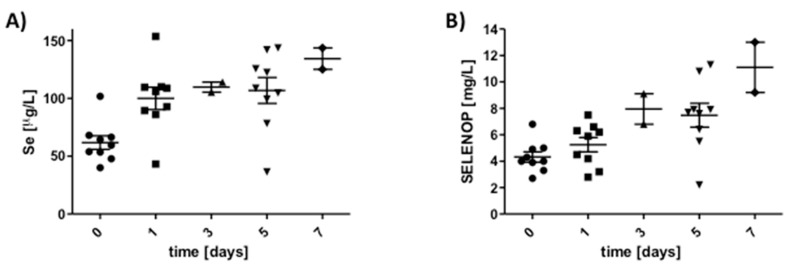
Time-resolved analysis of Se and SELENOP increase upon selenite infusion in cardiac surgery patients enrolled into the SOS-LVAD study. (**A**) Se concentrations increase in most of the cardiac patients undergoing elective heart surgery and selenite infusions (left); patient characteristics not yet de-blinded. (**B**) SELENOP concentrations increase in most of the patients in parallel to Se, partly reaching values of >7 or even >10 mg SELENOP/L after 5 or 7 d of selenite infusion (right).

The second cohort of samples was derived from patients undergoing elective cardiac surgery scheduled to undergo implantation of a ventricular assist device, which participated in a randomized controlled clinical trial of selenite supplementation. Plasma samples were taken before surgery (day 0), and at 1, 3, 5, and 7 days after the administration of the blinded investigational product. Total Se concentrations increased in the majority of patients during the time-period of analysis, partly reaching values exceeding 100 µg Se/L (Figure 5A). In parallel, SELENOP concentrations increased, and after five and seven days, three out of eleven samples displayed concentrations exceeding 10 mg SELENOP/L (Figure 5B). Under normal circumstances, similarly high SELENOP concentrations are not observed, and only 1 out of almost 2000 samples displayed a level of >10 mg SELENOP/L in a cross-sectional analysis of healthy European subjects, that is, with a frequency of less than 1 permille [11].

## 4. Discussion

**Figure 6:**
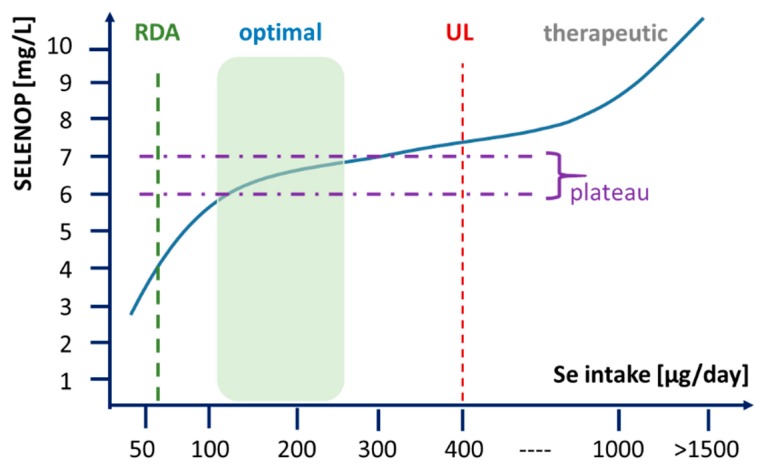
Circulating SELENOP concentrations as biomarkers of Se intake and Se status. Optimal supply with the essential trace element Se can be defined by reaching a plateau concentration of SELENOP in the range of 6–7 mg/L. This is not achieved by the current recommended dietary allowance (RDA), but requires higher intake in the range of 100–150 µg/day, depending on personal and environmental parameters (genotype, age, sex, health). Intervention studies regularly use supplemental Se in the dosage of up to 200 µg/day, that is, below the tolerable upper intake level (UL), resulting in concentrations still in the range of the saturated plateau of SELENOP expression. Current studies using therapeutic selenite concentrations in the range of several mg/day via i.v. application result in elevated SELENOP concentrations beyond the plateau, not observed before in clinical trials.

Our analyses were conducted in order to investigate whether circulating SELENOP concentrations may constitute a suitable biomarker of Se status in therapeutic application of high dosages of selenite in the clinical setting. We expected to observe maximal concentrations in the range of 5–7 mg SELENOP/L, as reported earlier from analytical supplementation studies [16,18,32]. To our surprise, SELENOP concentration increased beyond the presumed maximal value and even exceeded 10 mg/L in several patients receiving high dosages of selenite by i.v. administration. This result indicates that the maximal plasma levels of SELENOP described in prior studies may indicate a plateau of expression in response to intakes up to or around the UL (400 µg Se/d), but do not yet reflect the upper possible protein levels under an exceedingly high therapeutic Se supply. Our findings thus challenge the current concept on “saturation of SELENOP biosynthesis” at a maximal level upon a replete Se intake. Notably, our results are not in disagreement with the earlier studies, as no SELENOP analyses were ever conducted on samples from patients receiving selenite in dosages of >1 mg Se/d by i.v. application. We conclude that circulating SELENOP constitutes a biomarker for monitoring clinical trials with therapeutic dosages of supplemental selenite. Moreover, this notion may offer a new molecular explanation for the development of selenosis, that is, clinical signs of Se-related toxicity, by taking supraphysiological biosynthesis of SELENOP and SELENOP-dependent Se transport into target tissues into account that may develop upon excessive Se intakes, overcoming the plateau observed in prior studies of oral Se supplementation with lower dosages (Figure 6).

Two sets of clinical samples from independent supplementation trials formed the basis for this analysis. Both trials applied dosages of or exceeding 1 mg selenite/d, thereby exceeding the UL considerably. The rationale for applying these therapeutic dosages was based on several pre-clinical and analytical findings and considerations. In cancer, an Se deficit constitutes a risk factor for poor survival [33,34,35]. Cancer cells are in need of certain selenoproteins for their survival and proliferation, for example, certain GPX and thioredoxin reductase (TXNRD) isoenzymes [36]. Accordingly, tumors display the tendency of accumulating selenocompounds, causing increased intracellular Se concentrations that may act as cytotoxic agents [37,38]. It is thus conceivable to try to poison tumors by applying therapeutic amounts of Se that are within the window of the maximal tolerated dose [39]. Such a phase I study in terminally ill cancer patients was the *Selenite in the Treatment of Patients with Carcinoma* (SECAR) trial [27]. The dosages causing the first adverse effects amounted to several mg of selenite per square meter of body surface per day, and were much higher than anticipated. In comparison, the rationale for applying selenite in heart patients is different, as a severe Se deficit develops during cardiac surgery, resulting from myocardial ischemia/reperfusion and the following inflammatory response [40]. Under these conditions, a low Se status constitutes a risk factor for post-surgical complications and organ dysfunction [41]. Accordingly, intravenous therapeutic selenite supplementation prior to and during surgery is tested in an ongoing randomized clinical trial as a promising adjuvant measure for avoiding severe Se deficits aimed at improving surgical success and post-surgery course [9]. Both studies are in need of a Se-responsive biomarker for monitoring fate and anabolic effects of supplemental selenite. A measurement of total plasma Se concentrations would reflect successful i.v. application of selenite, that is, determine the drug applied, but would not be suitable to indicate metabolic fate or physiological effects of the supplemental trace element.

Circulating SELENOP seems to fulfill the requirements for a biomarker under these conditions. In both studies, the patients displayed baseline SELENOP concentrations below the European average, that is, below 4.4 mg SELENOP/L [11]. As expected, Se and SELENOP concentrations correlated tightly at the baseline, and increased in parallel in both studies, similar to the findings in other Se supplementation trials that monitored both biomarkers [16,18,19,20]. In the prior studies, saturation of SELENOP was observed when analyzing the dynamics of different dosages of supplemental selenomethionine [18], or when analyzing different Se-containing supplements ranging from 50 to 200 µg Se/d [16]. None of these studies applied dosages of selenite of 1.0 mg/d or greater, and accordingly, none observed resulting concentrations of 10 mg SELENOP/L or greater. Whether the elevated SELENOP concentrations observed in our analyses are the result of the choice of the intravenous application route, or the choice of using selenite as a supplemental selenocompound instead of Se-enriched yeast or selenomethionine, remains to be studied. Similarly, whether or not clinical success of the therapeutic Se applications is associated with an increase in SELENOP concentrations is unknown.

Changes in plasma SELENOP concentrations are transient following selenite supplementation, and the half-life of SELENOP may be in the range of 3–4 h, as determined with experimental rodents [42]. Under the assumption of a comparable stability of SELENOP in human blood, a continuous Se supplementation seems to be a prerequisite for maintaining the high plasma SELENOP levels intended in the clinical trials analyzed. On the other hand, the dynamic behavior of SELENOP concentrations is also of particular importance for a potentially toxic trace element like Se—a chronic excess of which may become harmful [43]. For these reasons, it appears advantageous to use selenite instead of selenomethionine in such clinical trials where a dynamic control of increasing and decreasing Se status is intended [44,45].

Among the strengths of our study are the analysis of longitudinal blood samples from two independent groups of patients receiving selenite dosages in very unusual amounts, and the parallel assessment of the two most informative biomarkers of Se status. Among the limitations are the relatively small groups of supplemented patients, the lack of a healthy control group, and the fact that both studies are not yet completed, thereby lacking additional clinical information.

## 5. Conclusions

The results indicate an unprecedented increase in plasma SELENOP concentrations in patients receiving i.v. injections of high therapeutic selenite dosages, beyond the threshold considered to constitute the absolute upper limit of circulating SELENOP. Hereby, SELENOP qualifies as an Se status biomarker for such studies, enabling the monitoring of therapeutic selenite administration and the anabolic effects of the supplemental Se on selenoprotein biosynthesis in the clinical setting.

## Supplementary Materials

The following are available online at https://www.mdpi.com/2072-6643/12/4/1067/s1, Figure S1: Individual responses of biomarkers of Se status to selenite infusion in the SECAR trial.

## References

[1] Labunskyy V.M., Hatfield D.L., Gladyshev V.N. (2014). Selenoproteins: Molecular pathways and physiological roles. Physiol. Rev..

[2] Combs G.F. (2015). Biomarkers of selenium status. Nutrients.

[3] Schomburg L., Schweizer U. (2009). Hierarchical regulation of selenoprotein expression and sex-specific effects of selenium. Biochim. Biophys. Acta.

[4] Vanderpas J.B., Contempre B., Duale N.L., Goossens W., Bebe N., Thorpe R., Ntambue K., Dumont J., Thilly C.H., Diplock A.T. (1990). Iodine and selenium deficiency associated with cretinism in northern zaire. Am. J. Clin. Nutr..

[5] Yu F.F., Han J., Wang X., Fang H., Liu H., Guo X. (2016). Salt-rich selenium for prevention and control children with kashin-beck disease: A meta-analysis of community-based trial. Biol. Trace Elem. Res..

[6] Loscalzo J. (2014). Keshan disease, selenium deficiency, and the selenoproteome. N. Engl. J. Med..

[7] Rayman M.P. (2012). Selenium and human health. Lancet.

[8] Wu Q., Rayman M.P., Lv H., Schomburg L., Cui B., Gao C., Chen P., Zhuang G., Zhang Z., Peng X. (2015). Low population selenium status is associated with increased prevalence of thyroid disease. J. Clin. Endocrinol. Metab..

[9] Benstoem C., Goetzenich A., Kraemer S., Borosch S., Manzanares W., Hardy G., Stoppe C. (2015). Selenium and its supplementation in cardiovascular disease—What do we know?. Nutrients.

[10] Hughes D.J., Duarte-Salles T., Hybsier S., Trichopoulou A., Stepien M., Aleksandrova K., Overvad K., Tjonneland A., Olsen A., Affret A. (2016). Prediagnostic selenium status and hepatobiliary cancer risk in the european prospective investigation into cancer and nutrition cohort. Am. J. Clin. Nutr..

[11] Hughes D.J., Fedirko V., Jenab M., Schomburg L., Meplan C., Freisling H., Bueno-de-Mesquita H.B., Hybsier S., Becker N.P., Czuban M. (2015). Selenium status is associated with colorectal cancer risk in the european prospective investigation of cancer and nutrition cohort. Int. J. Cancer.

[12] Steinbrecher A., Meplan C., Hesketh J., Schomburg L., Endermann T., Jansen E., Akesson B., Rohrmann S., Linseisen J. (2010). Effects of selenium status and polymorphisms in selenoprotein genes on prostate cancer risk in a prospective study of european men. Cancer Epidemiol. Biomark. Prev..

[13] Heller R.A., Seelig J., Bock T., Haubruck P., Grützner P.A., Schomburg L., Moghaddam A., Biglari B. (2019). Relation of selenium status to neuro-regeneration after traumatic spinal cord injury. J. Trace Elem. Med. Biol..

[14] Wiehe L., Cremer M., Wisniewska M., Becker N.P., Rijntjes E., Martitz J., Hybsier S., Renko K., Buhrer C., Schomburg L. (2016). Selenium status in neonates with connatal infection. Br. J. Nutr..

[15] Dumitrescu A.M., Liao X.H., Abdullah M.S.Y., Lado-Abeal J., Majed F.A., Moeller L.C., Boran G., Schomburg L., Weiss R.E., Refetoff S. (2005). Mutations in secisbp2 result in abnormal thyroid hormone metabolism. Nat. Genet..

[16] Hurst R., Armah C.N., Dainty J.R., Hart D.J., Teucher B., Goldson A.J., Broadley M.R., Motley A.K., Fairweather-Tait S.J. (2010). Establishing optimal selenium status: Results of a randomized, double-blind, placebo-controlled trial. Am. J. Clin. Nutr..

[17] Hoeflich J., Hollenbach B., Behrends T., Hoeg A., Stosnach H., Schomburg L. (2010). The choice of biomarkers determines the selenium status in young german vegans and vegetarians. Br. J. Nutr..

[18] Xia Y.M., Hill K.E., Li P., Xu J.Y., Zhou D., Motley A.K., Wang L., Byrne D.W., Burk R.F. (2010). Optimization of selenoprotein p and other plasma selenium biomarkers for the assessment of the selenium nutritional requirement: A placebo-controlled, double-blind study of selenomethionine supplementation in selenium-deficient chinese subjects. Am. J. Clin. Nutr..

[19] Hill K.E., Xia Y., Akesson B., Boeglin M.E., Burk R.F. (1996). Selenoprotein p concentration in plasma is an index of selenium status in selenium-deficient and selenium-supplemented chinese subjects. J. Nutr..

[20] Burk R.F., Norsworthy B.K., Hill K.E., Motley A.K., Byrne D.W. (2006). Effects of chemical form of selenium on plasma biomarkers in a high-dose human supplementation trial. Cancer Epidemiol. Biomark. Prev..

[21] Institute of Medicine (US) Panel on Dietary Antioxidants and Related Compounds (2000). Dietary Reference Intakes for Vitamin C, Vitamin E, Selenium, and Carotenoids.

[22] Huang Y., Wang Q.X., Gao J., Lin Z.Q., Banuelos G.S., Yuan L.X., Yin X.B. (2013). Daily dietary selenium intake in a high selenium area of enshi, china. Nutrients.

[23] Angstwurm M.W., Engelmann L., Zimmermann T., Lehmann C., Spes C.H., Abel P., Strauss R., Meier-Hellmann A., Insel R., Radke J. (2007). Selenium in intensive care (sic): Results of a prospective randomized, placebo-controlled, multiple-center study in patients with severe systemic inflammatory response syndrome, sepsis, and septic shock. Crit. Care Med..

[24] Forceville X., Laviolle B., Annane D., Vitoux D., Bleichner G., Korach J.M., Cantais E., Georges H., Soubirou J.L., Combes A. (2007). Effects of high doses of selenium, as sodium selenite, in septic shock: A placebo-controlled, randomized, double-blind, phase ii study. Crit. Care.

[25] Sakr Y., Maia V.P., Santos C., Stracke J., Zeidan M., Bayer O., Reinhart K. (2014). Adjuvant selenium supplementation in the form of sodium selenite in postoperative critically ill patients with severe sepsis. Crit. Care.

[26] Chelkeba L., Ahmadi A., Abdollahi M., Najafi A., Ghadimi M.H., Mosaed R., Mojtahedzadeh M. (2017). The effect of high-dose parenteral sodium selenite in critically ill patients following sepsis: A clinical and mechanistic study. Indian J. Crit. Care Med..

[27] Brodin O., Eksborg S., Wallenberg M., Asker-Hagelberg C., Larsen E.H., Mohlkert D., Lenneby-Helleday C., Jacobsson H., Linder S., Misra S. (2015). Pharmacokinetics and toxicity of sodium selenite in the treatment of patients with carcinoma in a phase i clinical trial: The secar study. Nutrients.

[28] Hybsier S., Schulz T., Wu Z., Demuth I., Minich W.B., Renko K., Rijntjes E., Kohrle J., Strasburger C.J., Steinhagen-Thiessen E. (2017). Sex-specific and inter-individual differences in biomarkers of selenium status identified by a calibrated elisa for selenoprotein p. Redox Biol..

[29] Ballihaut G., Kilpatrick L.E., Kilpatrick E.L., Davis W.C. (2012). Multiple forms of selenoprotein p in a candidate human plasma standard reference material. Metallomics.

[30] Kahaly G.J., Riedl M., Konig J., Diana T., Schomburg L. (2017). Double-blind, placebo-controlled, randomized trial of selenium in graves hyperthyroidism. J. Clin. Endocrinol. Metab..

[31] Burk R.F., Hill K.E. (2005). Selenoprotein p: An extracellular protein with unique physical characteristics and a role in selenium homeostasis. Annu. Rev. Nutr..

[32] Combs G.F., Jackson M.I., Watts J.C., Johnson L.K., Zeng H., Idso J., Schomburg L., Hoeg A., Hoefig C.S., Chiang E.C. (2012). Differential responses to selenomethionine supplementation by sex and genotype in healthy adults. Br. J. Nutr..

[33] Harris H.R., Bergkvist L., Wolk A. (2012). Selenium intake and breast cancer mortality in a cohort of swedish women. Breast Cancer Res. Treat..

[34] Meyer H.A., Endermann T., Stephan C., Stoedter M., Behrends T., Wolff I., Jung K., Schomburg L. (2012). Selenoprotein p status correlates to cancer-specific mortality in renal cancer patients. PLoS ONE.

[35] Lubinski J., Marciniak W., Muszynska M., Huzarski T., Gronwald J., Cybulski C., Jakubowska A., Debniak T., Falco M., Kladny J. (2018). Serum selenium levels predict survival after breast cancer. Breast Cancer Res. Treat..

[36] Stafford W.C., Peng X.X., Olofsson M.H., Zhang X.N., Luci D.K., Lu L., Cheng Q., Tresaugues L., Dexheimer T.S., Coussens N.P. (2018). Irreversible inhibition of cytosolic thioredoxin reductase 1 as a mechanistic basis for anticancer therapy. Sci. Transl. Med..

[37] Gundimeda U., Schiffman J.E., Gottlieb S.N., Roth B.I., Gopalakrishna R. (2009). Negation of the cancer-preventive actions of selenium by over-expression of protein kinase c epsilon and selenoprotein thioredoxin reductase. Carcinogenesis.

[38] Steinbrenner H., Speckmann B., Sies H. (2013). Toward understanding success and failures in the use of selenium for cancer prevention. Antioxid. Redox Signal..

[39] Misra S., Boylan M., Selvam A., Spallholz J.E., Bjornstedt M. (2015). Redox-active selenium compounds-from toxicity and cell death to cancer treatment. Nutrients.

[40] Stoppe C., Spillner J., Rossaint R., Coburn M., Schalte G., Wildenhues A., Marx G., Rex S. (2013). Selenium blood concentrations in patients undergoing elective cardiac surgery and receiving perioperative sodium selenite. Nutrition.

[41] Stoppe C., Schalte G., Rossaint R., Coburn M., Graf B., Spillner J., Marx G., Rex S. (2011). The intraoperative decrease of selenium is associated with the postoperative development of multiorgan dysfunction in cardiac surgical patients. Crit. Care Med..

[42] Burk R.F., Hill K.E., Read R., Bellew T. (1991). Response of rat selenoprotein p to selenium administration and fate of its selenium. Am. J. Physiol..

[43] Schomburg L. (2020). The other view: The trace element selenium as a micronutrient in thyroid disease, diabetes, and beyond. Hormones (Athens).

[44] Schomburg L., Kohrle J. (2008). On the importance of selenium and iodine metabolism for thyroid hormone biosynthesis and human health. Mol. Nutr. Food Res..

[45] Schrauzer G.N. (2003). The nutritional significance, metabolism and toxicology of selenomethionine. Adv. Food Nutr. Res..

