# Supplementary material for: Selenoprotein P as Biomarker of Selenium Status in Clinical Trials with Therapeutic Dosages of Selenite
Source: Nutrients. 2020 Apr 12;12(4):1067. doi: 10.3390/nu12041067 (PMC7230801; doi:10.3390/nu12041067)
Supplement: Supplementary file 1 [file nutrients-12-01067-s001.pdf]

Supplementary Figure S1:

Individual responses of biomarkers of Se status to selenite infusion in the SECAR trial

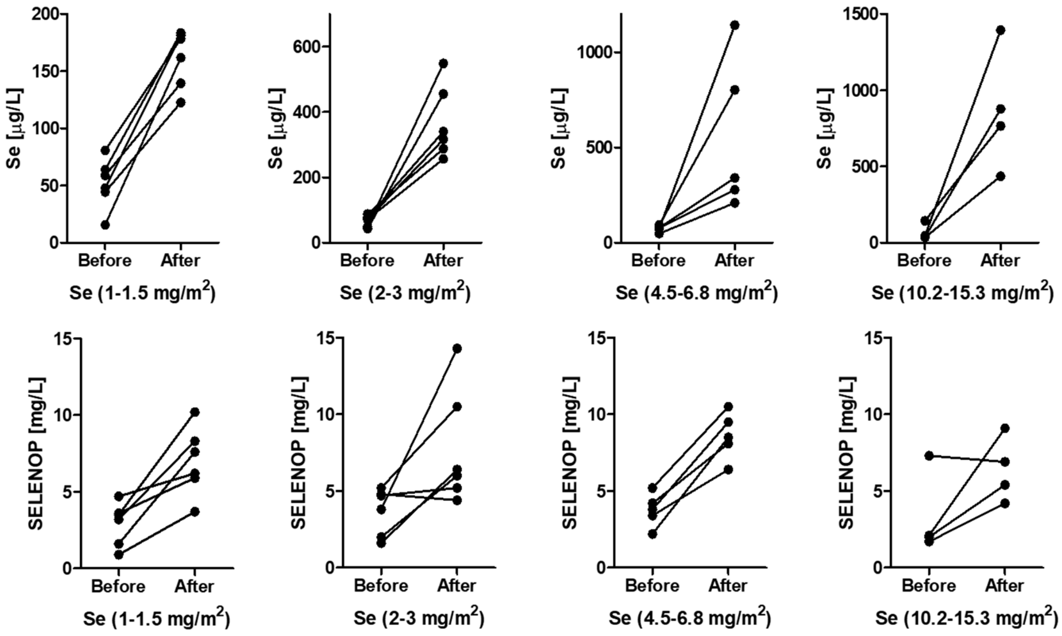

Supplementary Figure S1. Dynamics of SELENOP concentration increase during the first days of selenite treatment in the SECAR trial. Groups of patients received different amounts of Se (i.v.) ranging from 1 to 33.4 mg selenite/m^2. Plasma SELENOP concentrations were determined in the patients in relation to the applied Se dosages. All patients displayed increasing plasma Se concentrations. SELENOP increased similarly strongly in most patients, albeit not in all of the patients analyzed.
